# A Novel Digital PCR Assay for Accurate Detection and Differentiation of Focal and Non-Focal Subtypes of Mesenchymal–Epithelial Transition (*MET*) Gene Amplification in Lung Cancer

**DOI:** 10.3390/cancers17050811

**Published:** 2025-02-26

**Authors:** Raymond C. M. Shek, Peggy S. N. Li, Shelley C. M. Leung, H. T. Chu, F. Hioe, Victor W. L. Tang, Y. H. Lui, Larry R. S. Lam, Joshua H. Y. Ng, Raiden T. S. Wong, Miranda C. Y. Yau, Jimmy Y. W. Lam, Gilman K. H. Siu

**Affiliations:** 1 Department of Pathology, Pamela Youde Nethersole Eastern Hospital, Hospital Authority, Hong Kong, China; 2 Department of Health Technology and Informatics, The Hong Kong Polytechnic University, Hong Kong, China

**Keywords:** *MET* amplification, digital PCR, FISH, NGS, lung cancer, focal *MET* amplification, *MET* polysomy

## Abstract

Background/Objectives: Mesenchymal–epithelial transition (*MET*) gene amplification is a critical biomarker in non-small cell lung cancer (NSCLC), significantly influencing treatment decisions and prognostic evaluations. However, current detection methods such as fluorescence in situ hybridization (FISH) and next-generation sequencing (NGS) have limitations in speed, cost, and specificity, particularly when distinguishing between focal *MET* amplification and *MET* polysomy. Methods: This study introduces a novel digital PCR (dPCR) assay designed not only to detect *MET* amplification but also to differentiate between its focal and non-focal subtypes. The assay was evaluated against established FISH and targeted NGS panels using 55 NSCLC samples with known *MET* amplification statuses (26 positive and 29 negative) confirmed by FISH and NGS. Results The dPCR assay demonstrated high sensitivity (96.0%) and specificity (96.7%), achieving 100% concordance with FISH in differentiating focal *MET* amplification from *MET* polysomy. Additionally, the assay exhibited excellent precision, accuracy, and linearity (R^2^ = 1.00) in *MET* copy number quantification, surpassing NGS in diagnostic performance. Offering a robust, cost-effective, and efficient alternative to FISH, the dPCR assay significantly reduces the turnaround time (3 h versus 2 days) and provides a quantitative and objective method for *MET* amplification detection and subtype differentiation. This makes it suitable for clinical laboratories with limited molecular expertise. Conclusions: This study highlights the potential of the dPCR assay to complement existing molecular diagnostic techniques, delivering reliable and actionable results for *MET*-targeted therapy selection in NSCLC patients and thereby advancing precision oncology.

## Simple Summary

This study presents a novel digital PCR (dPCR) assay for detecting and differentiating focal and non-focal *MET* amplification in non-small cell lung cancer (NSCLC). Compared to fluorescence in situ hybridization (FISH) and next-generation sequencing (NGS), the dPCR assay offers superior diagnostic performance, with 96.0% sensitivity, 96.7% specificity, and 100% concordance with FISH in distinguishing focal *MET* amplification from *MET* polysomy. It also provides precise, accurate, and linear *MET* copy number quantification (R^2^ = 0.9951), outperforming NGS. The dPCR assay is faster (3 h vs. 2 days for FISH), cost-effective, and user-friendly, making it an ideal tool for clinical labs with limited molecular expertise. By delivering reliable and actionable results, this assay has the potential to complement existing diagnostic methods and support MET-targeted therapy selection, advancing precision oncology for NSCLC patients.

## 1. Introduction

Lung cancer is the most commonly diagnosed cancer and the leading cause of cancer-related mortality worldwide. It is estimated that there are nearly 2.5 million new cases (1 in 8 cancers) and 1.8 million deaths (1 in 5 deaths) globally [1]. Non-small cell lung cancer accounts for approximately 85% of all lung cancer cases [2,3]. While early-stage lung cancer patients have the best prognosis, the majority of patients are diagnosed with advanced or metastatic disease, resulting in a dismal 5-year survival rate of only 4% [4]. Precision medicine has emerged as a promising approach to improve outcomes for patients with lung cancer, with targeted therapies directed at specific genetic alterations playing a crucial role. The most well-known targetable gene alterations in lung cancer include mutations or amplifications in *EGFR* (10–15% of cases), *ALK* (2–7%), and *KRAS* (25–30%) [5,6]. However, an increasing number of less common but still clinically relevant genetic targets, such as *MET* (Mesenchymal–epithelial transition) (3–7% of cases), are being identified and incorporated into treatment guidelines [5]. 

*MET*, the proto-oncogene encoding the tyrosine kinase receptor for hepatocyte growth factor, has been found to be an important oncogenic driver in lung cancers, particularly in the adenocarcinoma subtype [7,8]. *MET* can be altered through various mechanisms, including gene amplification, mutations, and exon 14 skipping. *MET* amplification is estimated to occur in approximately 3–5% of non-small cell lung cancer cases, while *MET* mutations and exon 14 skipping are observed in 3–4% and 3–5% of cases, respectively [9]. This genetic alteration can lead to aberrant activation of the *MET* signaling pathway, promoting cell proliferation, survival, and metastasis. *MET* amplification can be classified into two distinct subtypes: focal *MET* amplification, where the *MET* gene is selectively amplified, and non-focal amplification, which occurs due to polysomy of chromosomal 7, which is sometimes referred to as *MET* polysomy. Importantly, *MET* amplification has been associated with sensitivity to *MET*-targeted therapies [10,11]. The focal subtype of *MET* amplification, where the *MET* gene is selectively amplified, has been identified as a potential mechanism of resistance to EGFR tyrosine kinase inhibitors. This is particularly relevant, as patients with the focal *MET* amplification subtype may benefit from treatment with *MET*-targeted therapies that can overcome the resistance conferred by this genetic alteration [12,13,14]. Accurately detecting and differentiating between focal and non-focal *MET* amplification is critical for guiding appropriate treatment selection and management for these patients, yet it presents a significant challenge [7]. Recognizing the clinical significance of *MET* amplification, leading oncology guidelines, such as the National Comprehensive Cancer Network (NCCN) and the European Society for Medical Oncology (ESMO), have recommended routine testing for *MET* alterations in patients with lung cancer [15]. 

The current common methods for *MET* amplification assessment in lung cancer, such as fluorescence in situ hybridization (FISH) and targeted next-generation sequencing panel (targeted NGS panel), have limitations. FISH analysis for *MET* amplification has been hampered by a lack of consensus in interpretation, as well as technical challenges that necessitate scoring by medical experts, particularly in cases with high tumor heterogeneity and limited tissue availability [16,17,18]. Targeted NGS panel, while more comprehensive in covering more actionable targets, can be costly and time-consuming, lacks well-defined cutoffs and cannot differentiate between focal and non-focal *MET* amplification [19,20,21]. Digital PCR (dPCR) has emerged as a promising technique with high sensitivity and specificity for the absolute quantification of nucleic acids. Previous studies have demonstrated the feasibility of using droplet digital PCR (ddPCR) to detect *MET* amplification in non-small cell lung cancer (NSCLC) patients with EGFR-TKI resistance [22,23,24]. However, there has not been a study applying dPCR to the detection and differentiation of focal and non-focal *MET* amplification in lung cancer using formalin-fixed paraffin-embedded (FFPE) tissue samples. Further research is needed to validate and optimize this approach not only for the accurate detection but also for the differentiation of focal and non-focal *MET* amplification in lung cancer.

To address these challenges, we have developed a novel dPCR assay at the Molecular Laboratory, Department of Pathology, Pamela Youde Nethersole Eastern Hospital, Hong Kong, that can accurately detect and discriminate between focal and non-focal *MET* amplification in lung cancer samples. This study will provide a streamlining workflow for the dPCR assay to make the assay more accessible and efficient, providing a simple user-friendly and cost-effective workflow for easy adoption by clinical laboratories, even for laboratories with limited expertise in molecular techniques.

## 2. Materials and Methods

### 2.1. Study Design

**Figure 1:**
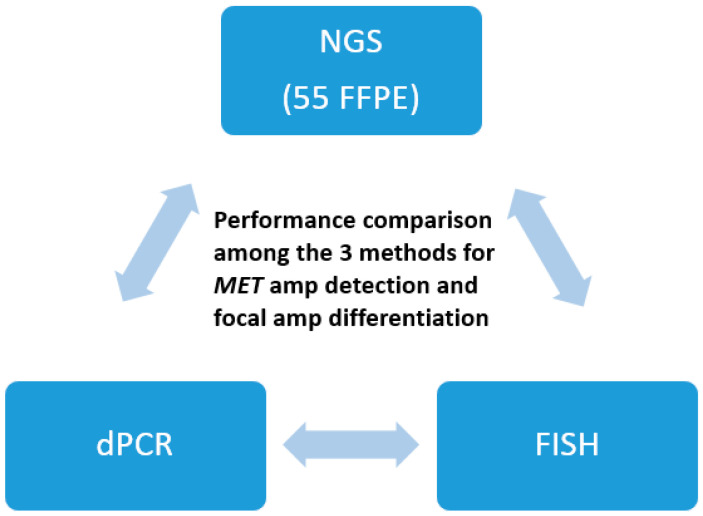
Overview of the study.

This is a retrospective study involving 55 lung cancer patients between Jan 2023 and Oct 2024. Lung tissues were obtained from all the patients, and targeted NGS panel results encompassing *MET* amplification status were obtained for all samples. There were in total 26 *MET* amplification-positive samples and 29 *MET* amplification-negative samples selected from the retrospective NGS results performed in the Molecular Laboratory, Pamela Youde Nethersole Eastern Hospital, Hong Kong. The samples selected were subsequently tested with our in-house dPCR assay and FISH to evaluate the performance of the dPCR assay in *MET* amplification detection and the differentiation of focal *MET* amplification and *MET* polysomy. In addition, a commercial standard (Seraseq™ Lung & Brain CNV Mix) was used to evaluate the linearity and precision of the *MET* copy number (CN) quantification. An overview of this study can be found in Figure 1. This study was approved by the Central Institutional Review Board (CIRB) of the Hospital Authority Hong Kong (Approval No. HKECREC-2021-053).

### 2.2. DNA Isolation from FFPE Tissue Samples

Nucleic acid was extracted from FFPE tissue samples using the EZ2 AllPrep DNA/RNA FFPE Kit (Qiagen, Hilden, Germany) following the manufacturer’s protocol. FFPE tissue sections of 10 µm thickness were cut and obtained, approximately totaling up to 2 mm^3^ of tissue. The sections were deparaffinized with the Paraffin Removal Solution (PRS) supplied in the EZ2 AllPrep DNA/RNA FFPE Kit at 56 °C for 3 min, followed by centrifugation at 20,000× *g* for 2 min. The supernatant was removed, the tissue pellet was resuspended by adding 150 µL of Buffer PKD and 10 µL of Proteinase K, and then the sample was incubated on a thermomixer at 56 °C for 15 min with shaking at 500 rpm. The supernatant was transferred to a new 1.5 mL microcentrifuge tube for RNA preparation, and the tissue pellet was kept for DNA preparation. Subsequently, 180 µL of Buffer ATL and 40 µL of Proteinase K were added to the tissue pellet, which was then overlaid with 200 µL of PRS. The mixture was loaded onto the EZ2 AllPrep DNA/RNA FFPE reagent cartridge, and the subsequent extraction was performed on the EZ2 Connect instrument (Qiagen, Hilden, Germany), with a 50 μL elution volume. The quantification of DNA extracts was performed following the manufacturer’s recommendations, using the Qubit^®^ dsDNA HS Assay Kit and the Qubit^®^ Fluorometer (Thermo Fisher Scientific, Inc., Waltham, MA, USA).

### 2.3. MET Amplification Detection Using FISH

**Figure 2:**
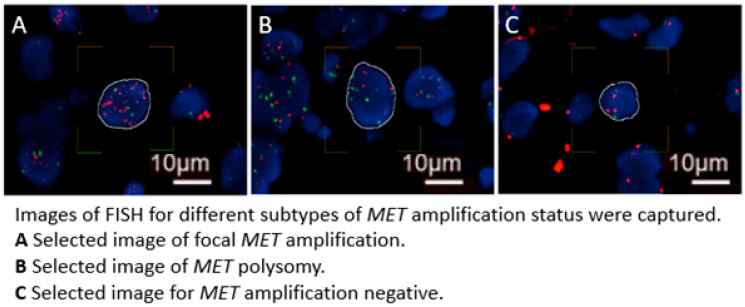
Images of FISH including the different subtypes of *MET* amplification status.

FISH analysis was performed on selected FFPE tissue sections to assess *MET* gene amplification. The tissue sections were first deparaffinized and then underwent hybridization with a *MET*/CCP7 Dual Color FISH Probe (CytoTest Inc., Rockville, MD, USA, catalog number CT-PAC014) according to the manufacturer’s instructions. The *MET* probe is labeled with red fluorescence, and it is designed to detect the human *MET* gene located on chromosome band 7q31.2. The CCP7 probe is labeled with green fluorescence, and it is designed to detect the centromere of chromosome 7 and is used for quantifying the number of chromosome 7 copies per cell. All FISH results were scored by qualified pathologists. For scoring, a total of 50 representative tumor cells from random areas with homogeneous *MET* signal distribution were selected, and the non-overlapping nuclei were examined across multiple fields on each slide. The mean copy number (CN) of each probe was recorded for all 50 cells, along with the percentage of cells exhibiting *MET* signal clusters and the percentage of cells with ≥5 copies of the *MET* signal. The *MET*/CCP7 ratio was calculated from the scores obtained from the overall scoring. Based on previous reports, *MET* amplification was defined as having a *MET* CN ≥ 5 and a *MET*/CCP7 ratio ≥ 2.0. In contrast, *MET* polysomy was characterized as having a *MET* CN ≥ 5 combined with a *MET*/CCP7 ratio < 2.0. Cases that did not meet these criteria were considered as *MET* amplification negative [13,25,26]. Images of FISH including the different subtypes of *MET* amplification status are shown in Figure 2.

### 2.4. MET Amplification Detection Using NGS

The extracted DNA samples were analyzed using the Thermo Fisher Scientific Oncomine Precision Assay GX (Thermo Fisher Scientific, Inc., Waltham, MA, USA) on the Ion Torrent Genexus Integrated Sequencer (Thermo Fisher Scientific, Inc., Waltham, MA, USA), following the manufacturer’s instructions. This assay enables simultaneous detection of hotspot mutations across 50 cancer driver genes, which includes *MET* amplification. The library preparation was performed in the automated Ion Torrent Genexus Integrated Sequencer, utilizing 10 ng of FFPE-extracted DNA as the input. Sequencing was conducted on the Ion Torrent GX5 chip, and the sequencing data were mapped to the hg19 reference genome. Subsequently, the sequencing data were analyzed using the Ion Torrent software (Genexus software V.6.8.1.1 Thermo Fisher Scientific). *MET* amplification was considered when the *MET* CN ≥ 5. All NGS results were reviewed and reported by qualified pathologists.

### 2.5. MET Amplification Detection Using dPCR in FFPE Samples

DNA extracted from FFPE tissue samples was tested using our in-house developed dPCR assay for detecting *MET* amplification. The assay determines the CN of the *MET* gene and a reference gene locus (REF1), specifically the CELF2 gene located on a different chromosome (chromosome 10), as well as the ratio between the *MET* gene and another reference gene locus (REF2), specifically the BRAF gene located on the same chromosome (chromosome 7). This approach allows for the detection of *MET* amplification and also for differentiation between focal *MET* amplification and *MET* polysomy. The primers and probes were designed using the NCBI Primer-BLAST tool to target highly conserved regions of the *MET*, *CELF2*, and *BRAF* genes. This design strategy minimizes the risk of ineffective binding due to sequence variations and enhances the specificity and sensitivity of the assay. The forward and reverse primers were optimized with a GC content of 50–60% and a melting temperature (Tm) of 58–60 °C, while the probe was designed with a slightly higher Tm (62–65 °C) to ensure efficient binding and to facilitate multiplexing within a single reaction. Cross-reactivity with non-target sequences was rigorously evaluated using in silico tools and experimental validation, confirming the absence of off-target binding. As a result, the primers and probes were verified to reliably amplify the target gene sequences, enabling the accurate quantification of *MET* copy number (CN) and precise differentiation between focal and non-focal *MET* amplification. Primer and probe sequences are listed in Table 1.

The dPCR assay was performed using the QIAcuity One 5-plex system (Qiagen, Hilden, Germany). The assay comprised two separate reaction mixes, with 20 ng of FFPE-extracted DNA used as input for each reaction. The master mix was prepared to a total volume of 40 µL and contained 10 µL of 4X concentrated QIAcuity Probe Mastermix (Qiagen, Hilden, Germany), 1.66 µL of each primer and probe mix, 20.68 µL of molecular-grade water, and 6 µL of sample DNA. The dPCR reactions were loaded into a QIAcuity Nanoplate 26k 24-well plate (Qiagen, Hilden, Germany) for PCR amplification. Reactions were partitioned into a maximum of 26,000 nanowells. The PCR conditions included an initial denaturation step at 95 °C for 2 min, followed by 40 cycles of 95 °C for 15 s (denaturation) and 56 °C for 30 s (annealing/extension). Following amplification, the Nanoplate was imaged on the QIAcuity One 5-plex system, and the data were analyzed using the QIAcuity Software Suite (version:2.5.0.1) (Qiagen, Hilden, Germany). The software assessed the number of wells with positive signals for the target genes, enabling subsequent calculations of *MET* copy number and differentiation of focal and non-focal amplification.

Reaction 1 contains primers and probes targeting the *MET* gene and REF1. This reaction is used to determine the *MET* gene copy number within the tumor cell fraction in the sample. Since the tissue samples contain both tumor and normal cells, and normal cells are assumed to have a diploid status, we derived a formula to provide a more accurate calculation of the *MET* gene copy number in the tumor cell fraction. The wildtype copy number (CN) for both genes was determined through the multiplication of the overall CN of REF1 obtained from dPCR by the wildtype cell percentage (WT%), specifically calculated as 100% minus the pathologist-scored tumor percentage. Subsequently, the tumor fraction CN for both the *MET* and REF1 genes was derived by subtracting the overall CN of both genes obtained in dPCR from the calculated wildtype CN. It is postulated that each tumor cell maintains a normal diploid status for the REF1 gene; therefore, the actual *MET* CN within an individual tumor cell was calculated by dividing the tumor fraction *MET* CN by the tumor fraction REF1 CN and multiplying by two. However, reaction 1 alone can only indicate the presence of *MET* gene amplification. It cannot differentiate between *MET* focal amplification and chromosome 7 polysomy.

Reaction 2 is the key for differentiating between *MET* focal amplification and non-focal *MET* amplification i.e., *MET* polysomy. It contains primers and probes targeting the *MET* gene and REF2. The reaction is intended for determining the ratio between the *MET* gene and REF2 on the same chromosome. If the *MET* amplification is focal, only the *MET* CN would increase, while the REF2 CN would remain at 2, giving rise to a ratio ≥ 2. If the *MET* amplification is due to chromosome 7 polysomy, both the *MET* and REF2 (BRAF gene) copy numbers would increase proportionally, maintaining a ratio close to 1. Data analysis was performed to calculate the tumor fraction CN for both the *MET* gene and REF2 and subsequently the ratio between them. The overall CN from dPCR was determined for both the *MET* gene and REF2. The wildtype CN for both genes was calculated by multiplying the overall CN of REF2 obtained from dPCR by the WT%. Subsequently, the tumor fraction CN for both the *MET* gene and REF2 was derived by subtracting the overall CN of both genes obtained from dPCR from the calculated wildtype CN. The tumor fraction *MET*/REF2 ratio was then calculated by dividing the tumor fraction *MET* CN by the tumor fraction REF2 CN. The formulas for analyzing the results of both reactions are summarized as below.

**(A)**  **Formula for MET CN analysis in a single tumor cell from reaction 1**
$$MET\;CN\;in\;a\;single\;tumor\;cell=\frac{MET-\left(100\%-T\%\right)*REF1}{T\%*REF1}*2$$

MET = Total dots of FAM signal in reaction 1

REF1 = Total dots of HEX signal in reaction 1

T% = Tumor percentage

**(B)**  **Formula for MET/REF2 ratio analysis from reaction 2**
$$MET/REF2\;ratio=\frac{MET-\left(100\%-T\%\right)*REF2}{T\%*REF2}$$

MET = Total dots of FAM signal in reaction 2

REF2 = Total dots of HEX signal in reaction 2

T% = Tumor percentage

The analyzed results from both reactions are considered together for interpretation and are summarized in Table 2. The *MET* amplification status is categorized into three subtypes: focal *MET* amplification, *MET* polysomy, and *MET* amplification negative. Focal *MET* amplification is defined as having a tumor fraction *MET* CN ≥ 5 in reaction 1 and simultaneously a tumor fraction MET/BRAF ratio ≥ 2 in reaction 2; *MET* polysomy is defined as having a tumor fraction *MET* CN ≥ 5 in reaction 1 but a tumor fraction MET/REF2 ratio < 2; *MET* amplification negative is defined as having a tumor fraction *MET* CN < 5 in reaction 1.

### 2.6. Statistical Analysis

The inter-run, intra-run precision, accuracy of CN calling, and linearity for *MET* CN quantification were calculated based on the tested results obtained using a commercial standard (Seraseq™ Lung & Brain CNV Mix). Linear Regression (R^2^) was calculated for assessing the consistency and linearity of the data. The performance of the dPCR assay in detecting *MET* amplification was evaluated by comparing the results to those obtained from the FISH and targeted NGS panel. The diagnostic performance (sensitivity, specificity, positive predictive value (PPV), and negative predictive value (NPV)) of the dPCR assay was established with respect to the orthogonal tests including FISH and targeted NGS panel on the selected clinical samples. The quantification of *MET* copy number by NGS, FISH, and dPCR was compared using paired *t*-tests. Statistical significance was assessed using two-sided *p*-values, with *p* ≤ 0.05 considered significant.

## 3. Results

### 3.1. Inter-Run/Intra-Run Precision, Accuracy of MET CN Calling, and Linearity for MET CN Quantification Using Commercial Standard

**Figure 3:**
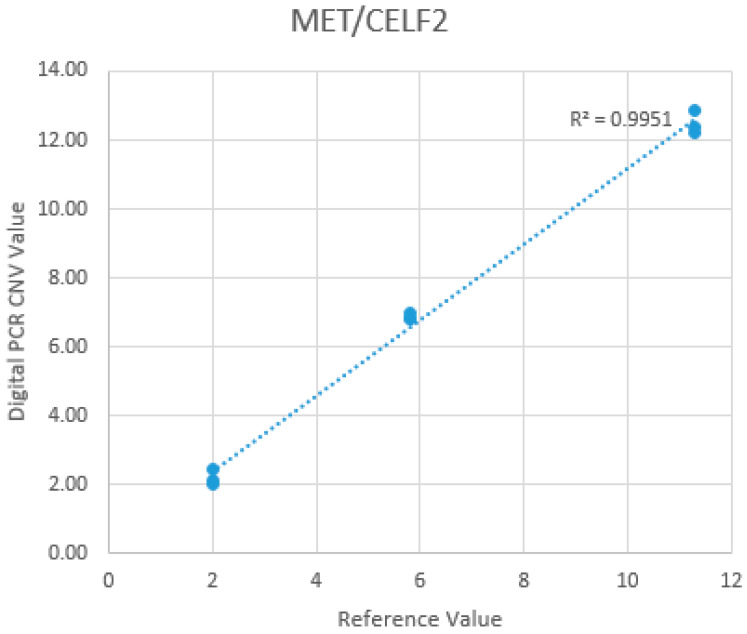
Correlation of dPCR *MET* CN quantification with reference.

The inter-run and intra-run precision, as well as the accuracy of *MET* CN calling and linearity for *MET* CN quantification, were evaluated at three different *MET* CN levels: +2 CN, +6 CN, and +12 CN. These *MET* CN levels were determined using commercially available DNA standards with known *MET* gene CNs (Seraseq™ Lung & Brain CNV Mix), as well as a normal sample verified to confirm the absence of *MET* amplification. All levels were tested in triplicate across three separate runs. The inter-run and intra-run precision were calculated based on the coefficient of variation (CV%) of the triplicate measurements at each CN level. The mean CNs obtained from the dPCR assay were 2.21 (range: 2.04–2.47; SD: 0.19; CV: 0.09; 95% CI) at the +2 CN level, 6.87 (range: 6.83–6.95; SD: 0.06; CV: 0.01; 95% CI) at the +6 CN level, and 12.47 (range: 12.19–12.85; SD: 0.27; CV: 0.02; 95% CI) at the +12 CN level. The mean CNs obtained for the three levels were in perfect correlation with the expected *MET* CNs, demonstrating the high precision and consistency of the *MET* CNs reported by the dPCR assay. For accuracy, the observed *MET* CNs by dPCR were 110.5%, 114.5%, and 103.9% of the expected values at the +2, +6, and +12 CN levels, respectively. Excellent linearity (R^2^: 0.9951) was also observed for the dPCR *MET* CN quantification (Figure 3).

### 3.2. Overview of Results Obtained from the NGS, dPCR, and FISH

**Figure 4:**
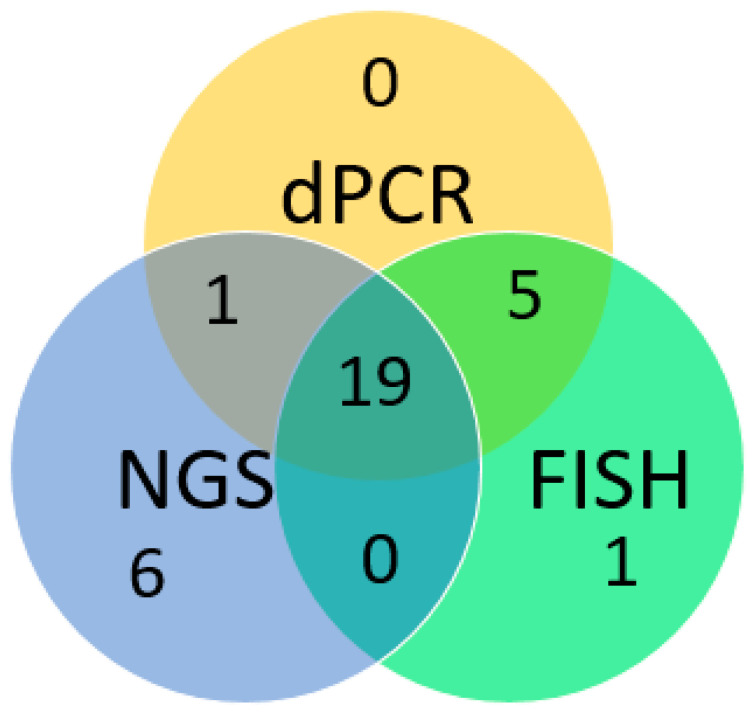
Concordance of the positive results obtained by dPCR, FISH, and NGS.

This study presents a comprehensive evaluation of the in-house dPCR for detection and classification of *MET* amplification against FISH and targeted NGS panel for FFPE tissues collected from 55 lung cancer patients. The overall results are summarized in Supplementary Materials. The concordance of the positive results obtained by dPCR, FISH, and NGS is shown in Figure 4.

### 3.3. Assessing the Correlation Between dPCR and FISH on MET Amplification Detection and Differentiation

**Figure 5:**
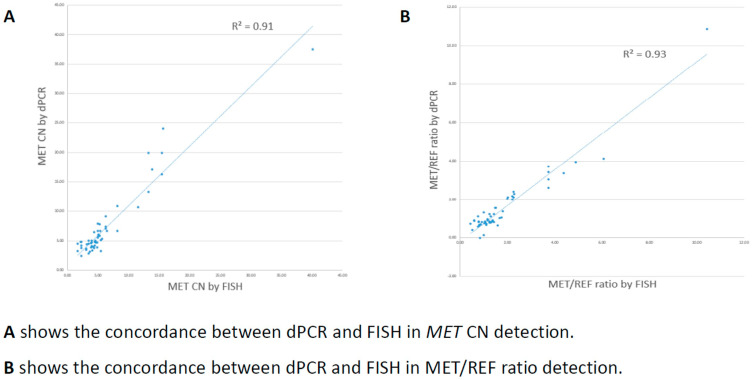
Concordance between dPCR and FISH.

Among the 55 samples selected, 25 samples were detected with *MET* amplification by FISH, 15 of which were interpreted as focal *MET* amplification (*MET* CN ≥ 5 and *MET*/CCP7 ratio ≥ 2) and 10 samples as *MET* polysomy (*MET* CN ≥ 5 and *MET*/CCP7 ratio < 2). The remaining 30 samples were FISH negative for *MET* amplification. Using FISH as the gold standard result for assessing the performance of the dPCR assay, 24 out of 25 FISH-positive cases were detected as *MET* amplification positive by dPCR. For focal *MET* amplification, all of the 15 FISH-positive cases were successfully detected and differentiated by dPCR, while for *MET* polysomy, 9 out of 10 FISH-positive cases were correctly detected. For FISH-negative cases, 29 out of 30 cases were correctly detected. Therefore, for *MET* amplification detection with reference to FISH results, the sensitivity, specificity, positive predictive value (PPV), and negative predictive value (NPV) were 96.0%, 96.7%, 96.0%, and 96.7%, respectively. For differentiation between focal *MET* amplification and *MET* polysomy, there was a 100% concordant rate between dPCR and FISH interpretation for all the dPCR-positive cases. Additionally, for CN quantification and *MET*/REF2 ratio calculation, dPCR and FISH showed a good linear association (R^2^ = 0.91 for CN quantification, *p* = 0.001; R^2^ = 0.93 for *MET*/REF2 ratio calculation) (Figure 5).

### 3.4. Comparison of the Performance Between dPCR and NGS for MET Amplification Detection

**Figure 6:**
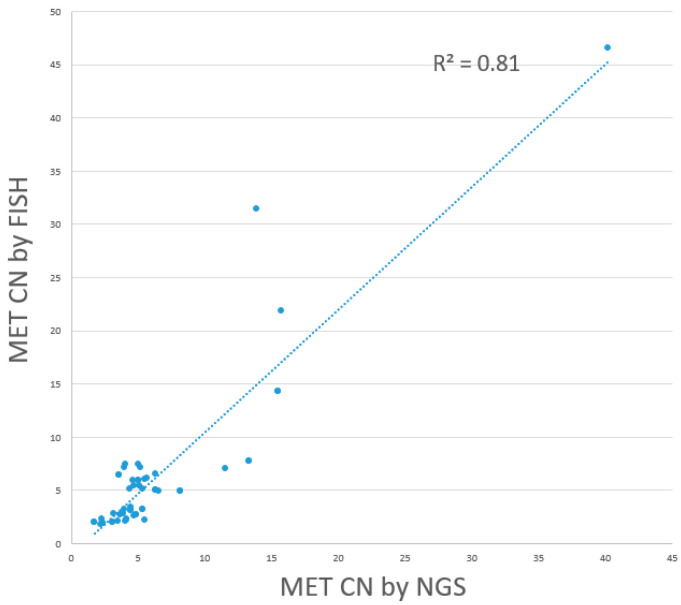
Concordance between NGS and FISH in *MET* CN detection.

In our current clinical practice, NGS is used for comprehensive gene panel testing of lung cancer patients. *MET* amplification is detected based on *MET* copy number variation (CNV), with a cutoff for NGS-positive *MET* amplification at *MET* CN greater than or equal to five. However, NGS serves primarily as a screening tool, and any detected *MET* amplification requires subsequent confirmation by FISH. An assessment of NGS performance in detecting *MET* amplification, compared to FISH as the gold standard, revealed that out of 25 FISH-positive cases, NGS detected 19 as *MET* amplification-positive, including 13 cases of focal *MET* amplification and 6 cases of *MET* polysomy. For the remaining 6 FISH-positive cases, NGS failed to detect the *MET* amplification. Among the 30 FISH-negative cases, 23 were also detected as *MET* amplification-negative by NGS, while 7 were detected as *MET* amplification-positive. The sensitivity, specificity, positive predictive value, and negative predictive value of NGS were 76.0%, 76.7%, 73.1%, and 79.3%, with a linear regression R^2^ value of 0.81 (*p* = 0.04), indicating a fair level of consistency (Figure 6). In comparison, the dPCR assay developed in this study demonstrated significantly better performance in terms of sensitivity, specificity, PPV, and NPV. The performance indicators of dPCR and NGS with reference to FISH, including the sensitivity, specificity, positive predictive value, and negative predictive value, are listed in Table 3 for comparison.

**Figure 7:**
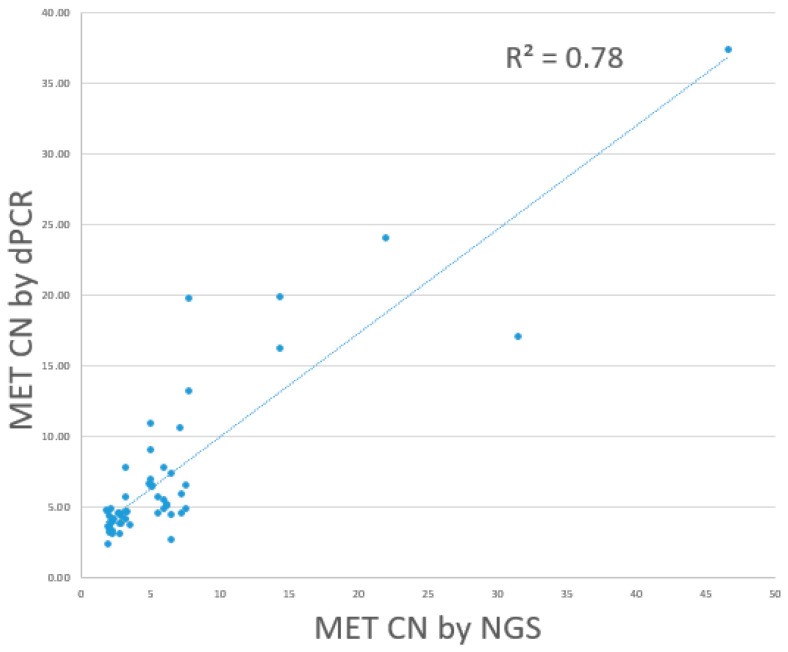
Concordance between dPCR and NGS in *MET* CN detection.

A direct comparison of the results of dPCR and NGS for *MET* amplification calling was performed. Comparison of the dPCR and NGS results on the 55 selected cases showed that 20 out of 26 NGS-positive cases were detected as *MET*-amplified by the dPCR assay, including 12 cases of focal *MET* amplification and 8 cases of *MET* polysomy. The remaining 6 NGS-positive cases were not detected as *MET*-amplified by the dPCR assay. Among the NGS-negative cases, 24 out of 29 were also classified as *MET* amplification-negative by the dPCR assay. The remaining 5 cases were detected as *MET* amplification-positive by dPCR. The positive percentage agreement, negative percentage agreement, and overall percentage agreement between dPCR and NGS were 79.9%, 82.8%, and 80%, respectively. Overall, dPCR and NGS showed a linear association. (R^2^: 0.78) (Figure 7).

## 4. Discussion

*MET* amplification has been associated with sensitivity to MET-targeted therapies. Growing evidence suggests that *MET* amplification is related to EGFR-TKI resistance and may contribute to acquired resistance in EGFR-mutated NSCLC patients treated with EGFR-TKIs. Accurately detecting and differentiating between focal and non-focal *MET* amplification is crucial for guiding appropriate treatment selection and management for these patients. In this study, we have developed and validated a novel digital PCR (dPCR) assay that enables the detection of *MET* amplification and the ability to differentiate between focal *MET* amplification and *MET* polysomy. Importantly, this study provides a comprehensive dataset consisting of results from NGS, dPCR, and FISH analyses, allowing for a thorough and objective performance comparison of these methodologies in detecting *MET* amplification.

The dPCR assay demonstrated high sensitivity and specificity of 96.0% and 96.7%, respectively. It can also provide accurate *MET* CN quantification comparable to the *MET* CN obtained from FISH, with a regression (R^2^) of 1.0. The dPCR also achieved a 100% concordance rate with FISH for the differentiation of focal *MET* amplification and *MET* polysomy. The results demonstrate that the dPCR assay can offer performance comparable to the gold standard FISH, yet it possesses several advantages over FISH. Though FISH is the current gold standard for *MET* amplification detection, it is a labor-intensive process that requires specialized medical expertise, as the FISH scoring must be performed by qualified pathologists. Additionally, the FISH result interpretation can be subjective due to variations in scoring area selection, tumor heterogeneity, tissue section quality, and nucleic acid preservation. In contrast, the dPCR assay is faster, easier, simpler, less expensive, and more objective in quantifying gene CN compared to FISH. The turn-around time (TAT) for the dPCR assay is around 3 h versus at least 2 days for FISH.

In addition to FISH, NGS is another emerging method for lung cancer molecular diagnostics. NGS enables comprehensive gene panel testing, allowing it to assess a broad range of genetic targets for diagnostic and therapeutic purposes. While targeted NGS panels excel at detecting hotspot mutations at low variant allele frequencies (VAFs), achieving accurate CNV calling, such as for *MET* amplification, can be challenging [27,28]. Factors such as varied amplicon region coverage, amplification biases and artifacts, poor DNA quality from FFPE samples, and limitations of computational alignment tools can undermine the reliability of gene-level CNV assessments by NGS. As a result, detecting *MET* amplification through amplicon-based NGS approaches may not be as reliable as the more robust FISH and dPCR methods. The findings of this study echo the above statements; the NGS-based assay showed a relatively lower sensitivity and specificity of 76.0% and 76.7%, respectively, in detecting *MET* amplification compared to the gold standard FISH method. The performance indicators, such as the sensitivity, specificity, PPV, and NPV, as well as the direct comparison between the dPCR and NGS results, demonstrated the superior performance of the dPCR assay over NGS in detecting *MET* amplification. Additionally, the dPCR assay can provide accurate differentiation between focal and non-focal amplification subtypes, further enhancing its clinical utility, while NGS cannot. The dPCR assay developed in this study is also easy to adopt in the clinical laboratory, and it has the potential to serve as a reliable, accurate, and cost-effective alternative to FISH and a supplementary tool to NGS gene panel results for *MET* amplification detection and discrimination in lung cancer patients.

While the dPCR assay demonstrated good performance, there were two discordant cases between the dPCR assay and the FISH results. In one case, the dPCR assay found the sample to be positive, while FISH found it to be negative. In the other case, the dPCR assay found the sample to be negative, but FISH found it to be positive. For the dPCR-positive and FISH-negative case, the dPCR assay reported a tumor fraction *MET* CN of 6.43, while the *MET* CN obtained from FISH was 4.38, which is marginally below the cutoff of 5. For the dPCR-negative and FISH-positive case, the dPCR assay reported a tumor fraction *MET* CN of 3.16, while the *MET* CN obtained from FISH was 5.53 with a *MET*/CCP7 ratio at 1.11 (less than 2), which is marginally above the cutoff of 5 and hence interpreted as marginally *MET* polysomy. This discrepancy could be due to several reasons. The tumor cellularity across different tissue sections may vary, so the FISH result may not be fully representative of the entire tumor sample. In contrast, the dPCR assay is tested on DNA extracted from multiple tissue sections. Additionally, tumor heterogeneity is commonly observed among the tumor cells in a single tissue section, leading to varied *MET* CN counts from different tumor cells. The *MET* CN obtained from FISH is the average of 50 selected tumor cells, while dPCR quantifies the overall *MET* CN in the DNA samples representing the entire tumor sample. When considering the NGS result for the same cases, the *MET* CN detected for the dPCR-positive and FISH-negative case was 5.13, while for the dPCR-negative and FISH-positive case, it was 2.21, which aligned with the interpretation of the dPCR results and were reported as marginally positive and negative, respectively. Therefore, the *MET* CN of this case is likely near the cutoff, and the discrepancy among the three testing methods is likely due to the differences in tumor cell abundance and genetic material heterogeneity. Despite these rare discordant cases, the overall performance of the dPCR assay was highly concordant with FISH, demonstrating its robust and reliable capability in detecting and differentiating *MET* amplification subtypes. The dPCR approach provides a more quantitative and objective assessment of *MET* copy numbers, which can complement the FISH results and help resolve challenging cases. These discordant cases also highlight the challenges in accurately detecting gene amplification, especially near the cutoff CN level, and the importance of utilizing complementary techniques such as dPCR, FISH, and NGS to reach a reliable diagnosis. Therefore, FISH, while considered the gold standard, is not a perfect technique, and its results can be influenced by subjective interpretation, sample quality, and technical variations. The scoring of FISH signals by pathologists can be challenging, especially in samples with low-level amplification or tumor heterogeneity. This subjectivity in FISH interpretation may contribute to occasional discrepancies between FISH and other more quantitative techniques such as dPCR. Furthermore, the dPCR assay developed in this study relies on the quantification of *MET* gene copies relative to a reference gene. While the assay demonstrated excellent linearity and precision, there may be rare instances where the reference gene copy number is also altered, leading to a skewed *MET* copy number assessment by dPCR.

Another matter to highlight is that the use of FFPE-derived DNA in this assay is critical for clinical translation, as FFPE remains the cornerstone of routine histopathological and molecular diagnostics. By demonstrating robust performance on FFPE samples, despite their inherent challenges of DNA fragmentation and degradation, this dPCR assay ensures compatibility with standard clinical workflows, enabling rapid integration into existing diagnostic pipelines.

There are several limitations of this study. The sample size is relatively small due to the limited samples collected within the study period, impacting the ability to establish an accurate cutoff value for *MET* amplification calling. The current cutoff is based on general FISH standards. With a larger sample size, a more comprehensive comparison between dPCR, FISH, and NGS could be conducted, refining the cutoff value using Receiver Operating Characteristic (ROC) analysis. Based on our current data, adjusting the cutoff to a CN value between 4.5 and 5 may enhance the concordance between dPCR and orthogonal tests. Additionally, this study did not assess the clinical utility of the dPCR assay in relation to treatment outcomes. Furthermore, exploring the assay’s performance on alternative dPCR platforms could enhance its adaptability for varied clinical laboratory settings.

In summary, the novel dPCR assay developed in this study offers a rapid, cost-effective, and objective method for detecting *MET* amplification and differentiating focal amplification from polysomy, addressing key limitations of FISH and NGS. The dPCR assay significantly reduces the turnaround time (TAT) to approximately 3 h, compared to the 2 days required for FISH and NGS, enabling timely clinical decision-making for MET-targeted therapies. Furthermore, dPCR is the most cost-effective option, with reagent costs of approximately USD 60 per test, compared to USD 250 per test for FISH and USD 650 per test for NGS. The lower cost of dPCR is attributed to its simplified workflow, lower reagent cost, reduced hands-on time, and the use of more affordable equipment. Additionally, dPCR requires less specialized expertise and infrastructure compared to FISH and NGS, further contributing to its cost efficiency. The dPCR assay demonstrates 100% concordance with FISH in distinguishing focal *MET* amplification (a therapeutically actionable biomarker) from polysomy (non-actionable), a critical distinction for therapy selection. This specificity surpasses NGS, which struggles with accurate copy number variation (CNV) detection due to technical biases. Importantly, only dPCR and FISH can differentiate focal from non-focal amplification, a capability essential for guiding treatment decisions. While rare discordant cases near the *MET* CN cutoffs underscore the value of complementary testing with FISH or NGS in ambiguous scenarios, the assay’s simplicity, efficiency, and cost-effectiveness make it well-suited for integration into routine clinical workflows. Future studies should focus on validating the assay in larger cohorts, refining the *MET* CN thresholds, and correlating the results with therapeutic outcomes to fully establish its clinical utility. By enabling timely and accurate identification of MET-driven resistance, this assay has the potential to optimize treatment strategies and improve patient outcomes in the era of precision oncology.

## 5. Conclusions

In conclusion, this study demonstrates the robustness and reliable performance of our in-house developed dPCR assay. The assay accurately detects and differentiates *MET* amplification subtypes, quantifies *MET* CN with high precision, and shows good concordance with the current gold standard FISH technique. It is considered to be a valuable complement to FISH and NGS testing for *MET* amplification assessment in clinical practice, offering advantages such as cost-effectiveness, a faster turnaround time, and reduced sample requirements as a comprehensive molecular diagnostic tool for lung cancer patients in clinical use.

## Figures and Tables

**Table 1:** Sequences of the primers and probes used in the dPCR assay.

Target Gene	Primer/Probe	Primer/Probe Sequence
*MET*	MET_Forward	5′-GACGGACCAGTCCTACATTGA-3′
	MET_Reverse	5′-CTAGAGTTTCCCTTTGGACCG-3′
	MET_Probe	5′-FAM-CTTACCCCATTAAGTATGTCCATGCCTTTG-MGB-3′
*CELF2* (REF1)	REF1_Forward	5′-AGAGGTTAACTTGGTGGCCT-3′
	REF1_Reverse	5′-AAAACAAGCCGATGTAGTGGA-3′
	REF1_Probe	5′-HEX-AGAAGCCAGGAGAAGCACTTACTCCAA-MGB-3′
*BRAF* (REF2)	REF2_Forward	5′- AATAGAGTCCGAGGCGGG-3′
	REF2_Reverse	5′- CCAATACCACAGGAAGAGGC-3′
	REF2_Probe	5′-HEX-GGATGATCCAGATGTTAGGGCAGTCTCT-MGB-3′

**Table 2:** Result interpretation for the dPCR assay.

Interpretation	*MET* Copy Number in Reaction 1	*MET*/*BRAF* Ratio in Reaction 2
Focal *MET* amplification	≥5	≥2
*MET* polysomy	≥5	<2
*MET* amplification negative	<5	N/A

**Table 3:** Comparison between dPCR and NGS with reference to FISH for *MET* amplification detection.

Method	Sensitivity	Specificity	PPV	NPV	Linear Regression (R^2^)
dPCR	96.0%	96.7%	96.0%	96.7%	0.91
NGS	76.0%	76.7%	73.1%	79.3%	0.81

## Data Availability

The original contributions presented in this study are included in the article/Supplementary Materials. Further inquiries can be directed to the corresponding author(s).

## Supplementary Materials

The following supporting information can be downloaded at: https://www.mdpi.com/article/10.3390/cancers17050811/s1.
